# MicroRNA Expression Profiling in Newcastle Disease Virus-Infected DF-1 Cells by Deep Sequencing

**DOI:** 10.3389/fmicb.2019.01659

**Published:** 2019-07-23

**Authors:** Yu Chen, Wen Liu, Haixu Xu, Jingjing Liu, Yonghuan Deng, Hao Cheng, Shanshan Zhu, Yuru Pei, Jiao Hu, Zenglei Hu, Xiaowen Liu, Xiaoquan Wang, Min Gu, Shunlin Hu, Xiufan Liu

**Affiliations:** ^1^ Animal Infectious Disease Laboratory, College of Veterinary Medicine, Yangzhou University, Yangzhou, China; ^2^ Jiangsu Co-innovation Center for Prevention and Control of Important Animal Infectious Diseases and Zoonosis, College of Veterinary Medicine, Yangzhou University, Yangzhou, China; ^3^ Jiangsu Key Laboratory of Zoonosis, College of Veterinary Medicine, Yangzhou University, Yangzhou, China

**Keywords:** miRNAs’ expression profile, NDV, deep sequencing, DF-1 cells, gga-miR-451, YWHAZ

## Abstract

Newcastle disease virus (NDV), causative agent of Newcastle disease (ND), is one of the most devastating pathogens for poultry industry worldwide. MicroRNAs (miRNAs) are non-coding RNAs that regulate gene expression by regulating mRNA translation efficiency or mRNA abundance through binding to mRNA directly. Accumulating evidence has revealed that cellular miRNAs can also affect virus replication by controlling host-virus interaction. To identify miRNA expression profile and explore the roles of miRNA during NDV replication, in this study, small RNA deep sequencing was performed of non-inoculated DF-1 cells (chicken embryo fibroblast cell line) and JS 5/05-infected cells collected at 6 and 12 h post infection (hereafter called mock‚ NDV-6 h, and NDV-12 h groups respectively). A total of 73 miRNAs of NDV-6 h group and 64miRNAs of NDV-12 h group were significantly differentially expressed (SDE) when compared with those in mock group. Meanwhile, 50 SDE miRNAs, including 48 up- and 2 down-regulated, showed the same expression patterns in NDV-6 h and NDV-12 h groups. qRT-PCR validation of 15 selected miRNAs’ expression patterns was consistent with deep sequencing. To investigate the role of these SDE miRNAs in NDV replication, miRNA mimics and inhibitors were transfected into DF-1 cells followed by NDV infection. The results revealed that gga-miR-451 and gga-miR-199-5p promoted NDV replication while gga-miR-19b-3p and gga-miR-29a-3p inhibited NDV replication. Further function research demonstrated gga-miR-451 suppressed NDV-induced inflammatory response *via* targeting YWHAZ (tyrosine3-monooxygenase/tryptophan5-monooxygenase activation protein zeta). Overall, our study presented a global miRNA expression profile in DF-1 cells in response to NDV infection and verified the roles of some SDE miRNAs in NDV replication which will underpin further studies of miRNAs’ roles between the host and the virus.

## Introduction

Newcastle disease (ND), a highly contagious disease caused by virulent Newcastle disease virus (NDV), is one of the most severe avian diseases and can cause significant economic loss for the global poultry industry (Alexander, 2000). Since its first outbreak in Indonesia in 1926, NDV has already caused at least four panzootics and still remains prevalent worldwide (Alexander, 2000). Previous studies have shown that a wide range of domestic and wild birds (at least 250 species) can be naturally or experimentally infected with NDV (Ganar et al., 2014). Although NDV belongs to a single serotype, it has a high genetic diversity and rapid evolution, which leads to failure in ND vaccine immunization or reduction of vaccine efficiency (Aldous and Alexander, 2001).

NDV is an enveloped virus with a negative-sense, single-stranded RNA genome of about 15 kb that contains six genes in the order of 3′-NP-P-M-F-HN-L-5′, encoding six main proteins (nucleoprotein, phosphor protein, matrix protein, fusion protein, hemagglutinin-neuraminidase, and large protein, respectively) (de Leeuw and Peeters, 1999). Like most paramyxoviruses, the P gene of NDV encodes the V and W proteins in addition to the P protein *via* RNA editing (Samson et al., 1991; Steward et al., 1993). Innate immunity acts as the first line of host defense against virus infection by recognizing NDV infection *via* pattern recognition receptors (PRRs) like RIG-I (retinoic-acid inducible gene I) (Kato et al., 2006; Sun et al., 2013), MDA5 (melanoma differentiation-associated) (Rue et al., 2011), and TLR3 (Toll-like receptor 3) (Cheng et al., 2014). Activated PRRs lead to the production of type I IFNs (interferon) and inflammatory cytokines to limit NDV replication. However, during the constant combat between the virus and host immune system, NDV has also evolved some mechanisms to antagonize host antiviral responses. For example, the V protein of NDV can inhibit IFN-beta promoter activation by binding to MDA-5 (Andrejeva et al., 2004) and also can antagonize IFN response by degrading STAT1 to suppress Janus kinase/signal transducers and activators of transcription (JAK-STAT) pathway (Qiu et al., 2016).

MiRNAs, composed of 21–23 nucleotides, are endogenous small non-coding RNAs and can negatively regulate expression level of target genes through either mRNA degradation or translational repression (Bartel, 2004; Xiao and Rajewsky, 2009). Recent studies demonstrate that cellular miRNAs are capable of regulating virus translation and replication by direct binding to virus genome or through virus-mediated changes in the host transcriptome (Trobaugh and Klimstra, 2017). Similarly, virus infection can also in turn mediate changes in expression profiles of host miRNAs, which can greatly influence viral life cycles, viral tropism, and pathogenesis of viral diseases (Ghosh et al., 2009; Skovgaard et al., 2013; Slonchak et al., 2014; Liu et al., 2015). For instance, miR-451 has been identified as a key factor involved in regulating the expression of inflammatory cytokines during influenza virus (Rosenberger et al., 2012) and *Mycoplasma gallisepticum* infection (Zhao et al., 2018) *via* targeting YWHAZ. In addition, miR-146a was elevated in the blood of ferrets and horses infected with hendra virus (HeV), and this miRNA targeted RNF11 (ring finger protein 11) mRNA and facilitated HeV replication (Stewart et al., 2013). Moreover, the mechanisms of miRNA regulating viral infection and replication were characterized for many viruses, such as human immunodeficiency virus (HIV) (Huang et al., 2007; Nathans et al., 2009), vesicular stomatitis virus (VSV) (Otsuka et al., 2007), hepatitis C virus (HCV) (Chowdhury et al., 2012; Mukherjee et al., 2015; Bandiera et al., 2016), respiratory syncytial virus (RSV) (Bakre et al., 2012; Thornburg et al., 2012), Nipah virus (NiV) (Foo et al., 2016), and Japanese encephalitis virus (JEV) (Zhu et al., 2015; Sharma et al., 2016). Although host miRNAs play a crucial role in regulating viral infection and replication, there is still limited report about the function of host miRNAs in the process of NDV infection. Harshad Ingle et al. have shown that the expression of hsa-miR-485 in human embryonic kidney (HEK) 293 T cells was induced in response to NDV infection and this miRNA targeted RIG-I mRNA directly for degradation, which led to suppression of antiviral response and enhanced NDV replication (Ingle et al., 2015). Integrated analysis of miRNA and mRNA expression profiles in NDV-infected chicken embryonic visceral tissues revealed that 130 miRNA-mRNA pairs were involved in the regulation of immunity or inflammatory responses (Jia et al., 2018). These results suggested that cellular miRNAs play an important role in host antiviral response against NDV infection. Despite these findings, the molecular mechanism of miRNAs’ function during NDV infection in avian cells is still largely unknown.

In the present study, DF-1 cells were inoculated with NDV JS5/05 strain, and miRNA expression profiles of mock, NDV-6 h, and NDV-12 h groups were analyzed. Fifty SDE miRNAs showed the same expression patterns in NDV-6 h and NDV-12 h groups when compared with mock group. Moreover, gga-miR-451 and gga-miR-199-5p promoted virus replication, whereas gga-miR-19b-3p and gga-miR-29a-3p suppressed virus replication. Additionally, gga-miR-451 negatively regulated inflammatory response in NDV-infected DF-1 cells by targeting YWHAZ and subsequently inhibited NDV replication.

## Materials and Methods

### Virus and Cells

Velogenic genotype VII NDV strain JS5/05 (Accession Number: JN631747) was propagated in chicken embryos and the biological characteristics of the virus were determined previously (Hu et al., 2011). Velogenic genotype IV NDV strain Herts/33(Accession Number: AY741404) was obtained from Dr. D. J. Alexander (Animal Health and Veterinary Laboratories Agency, UK). Velogenic genotype IX NDV strain F48E8 (Accession Number: FJ436302), avirulent genotype I LX (Accession Number: KF494201), and genotype II La Sota (Accession Number: AF077761) were isolated and maintained in our laboratory. DF-1 cells(ATCC CRL-12203) were cultured in Dulbecco’s modified Eagle’s medium (DMEM) (Life Technologies, USA) supplemented with 10% fetal bovine serum (FBS) (Life Technologies, USA), 100 U/ml penicillin, and 100 μg/ml streptomycin at 37°C under 5% CO_2_ atmosphere.

### Viral Infection and RNA Isolation

DF-1 cells were seeded at a density of 1 × 10^6^ per well in six-well cell culture plates (Corning, NY, USA). After 24 h, cell monolayers with 80–90% confluency were washed three times with phosphate-buffered saline (PBS) and then inoculated with NDV strain JS5/05 at a multiplicity of infection (MOI) of 0.1. After 1 h of adsorption, the culture medium was replaced with fresh medium containing 1% FBS. Cells in three non-inoculated or virus-infected wells were trypsinized with 0.5 ml of trypsin (2 μg/ml) (Sigma, Santa Clara, USA) at 0, 6, and 12 h post infection (hpi) respectively and centrifuged at 1200 *g* for 8 min and washed three times with ice-cold PBS. Next, the DF-1cells from three wells were pooled separately for subsequent total RNA extraction using the EasyPure RNA kit (TransGen Biotech, Nanjing, China) according to the manufacturer’s instructions. The total concentration and RNA quality of the samples were measured with a Nano Drop ND-2000 spectrophotometer (Thermo Fisher Scientific, Waltham, USA) and an Agilent 2200 Bioanalyzer (Agilent Technologies, Santa Clara, USA). RNA integrity was also evaluated by agarose gel electrophoresis with 1.5% agarose gel and 135 V for 25 min.

### Library Construction and Small RNA Deep Sequencing

For small RNA library construction, proprietary adapters were first ligated to the 5′ and 3′ ends of the total RNAs, and then these adapters-ligated samples were used as templates for synthesis of the first strand of cDNA. Subsequently, cDNA was amplified by 16 PCR cycles to enrich the libraries. Following the enrichment, the PCR products about 150 bp in length were purified by polyacrylamide gel electrophoresis, the quality and concentrations were measured with an Agilent 2200 Bioanalyzer. The DNA fragments in the libraries were eventually used for sequencing on an Illumina HiSeq 2500 instrument (Illumia Inc., San Diego, USA) according to the manufacturer’s instructions.

### Analysis of Deep Sequencing Data

The 50 nt sequence tags from the deep sequencing were filtered by removing the 5′ and 3′ adapters sequences, low-quality reads, and redundancy contaminants to obtain the clean reads. After quantity control program, the clean reads were mapped to *Gallus gallus* genome using Burrows-Wheeler Alignment Tool (BWA) and their expression and distribution patterns were analyzed using the Short Oligonucleotide Alignment Program (SOAP) (Li et al., 2008). Next, the clean reads were classified and annotated into different categories by screening against authoritative databases including miRBase version 21.0 database^1^, Rfam 12.1 database^2^, and piRNA bank database^3^. The quantity of known miRNAs is normalized and showed as reads per million (RPM) using the following computational formula: RPM = (number of reads mapping to miRNA/number of reads in clean data) × 10^6^. To determine the effects of NDV infection on cellular miRNA expression, the known miRNA expression levels of NDV-6 h and NDV-12 h groups were compared with mock group using edgeR package (Robinson et al., 2010). *p* <0.05 was considered as a significant difference. │log_2_ (fold change)│ > 1 was set as the default threshold for significantly differential expression.

### Target Prediction and Functional Enrichment of Differentially Expressed miRNAs

MiRanda^4^, PITA^5^, and RNAhybrid^6^ algorithms were used to predict the target genes of 50 common SDE miRNAs. The results were combined and the overlaps were designed as the predicted miRNA target genes. Subsequently, these genes were functionally annotated through the cell component, biological process, and molecular function information supported by gene ontology (GO)::Termfinder software. The value of *p* was calculated using the hypergeometric distribution and corrected by false discovery rate (FDR) (Boyle et al., 2004). Meanwhile, these target genes were submitted to Kyoto Encyclopedia of Genes and Genomes (KEGG) pathways^7^ analysis. We used scripts in house to enrich significant differential expression gene in KEGG pathways. The value of *p* was calculated through Fisher’s Exact Test and corrected by FDR. GO terms and KEGG pathways with corrected *p*<0.05 were considered to represent significant enrichment.

### Stem-Loop qRT-PCR of miRNAs

MiRNA Extraction Kit was used to extract miRNAs from the mock, NDV-6 h, and NDV-12 h groups according to manufacturer’s instructions. To verify miRNA expression levels in each sample, cDNA synthesis was carried out according to the protocol of One Step miRNA cDNA Synthesis Kit, in which polyA tailing of the miRNAs was followed by reverse transcription with a tagged polyT primer described elsewhere (Balcells et al., 2011). Briefly, the reaction mix (20 μl) consisting of 100 ng of miRNA, 5 μl of 4 × One step miRNA RT Solution, and 2 μl of 10 × miRNA RT Primer was incubated at 37°C for 1 h followed by enzyme inactivation at 95°C for 5 min. Primers used to detect miRNA expression levels are shown in Table 1. Quantification of miRNA was performed by HG miRNA SYBR Green PCR Kit. qRT-PCR was performed in 20-μl volume composed of 1 μl of cDNA, 1 μl of each primer, and 4μl of Golden HS SYBR Green qPCR Mix under the following conditions: 95°C for 15 min, 35 cycles at 95°C for 5 s, and 60°C for 30s in LightCycler 480 (Roche, Basel, Switzerland). All kits used for miRNA extraction and qRT-PCR were purchased from HaiGene, Harbin, China. To measure the dynamic expression pattern of gga-miR-451 after NDV infection, DF-1 cells were infected with NDV strain JS5/05 at an MOI of 0.1 for indicated times or at an indicated MOI for 12 h and then miRNAs were extracted and gga-miR-451 were detected by stem-loop qRT-PCR as described above. MiRNA expression levels were normalized to 5s rRNA levels using the 2^−ΔΔCt^ model.

**Table 1:** Primers used to detect miRNA expression levels with qRT-PCR.

Gene	Forward primer (5′–3′)	Reverse primer (5′–3′)
RT primer	GTCGGTGTCGTGGAGTCGTTTGCAATTGCACTGGATTTTTTTTTTTTTTTTTTV	
gga-miR-130a-5p	CAGGCCCTTTTTCTGTTGT	AGGTCCAGTTTTTTTTTTTTTTTAGTAG
gga-miR-1434	GCAGGTGCGTGATGATG	GGTCCAGTTTTTTTTTTTTTTTAATTTTCC
gga-miR-199-5p	CCCAGTGTTCAGACTACCTG	GTCCAGTTTTTTTTTTTTTTTGAACAG
gga-miR-301a-3p	GCAGCAGTGCAATAATATTGTCA	TCCAGTTTTTTTTTTTTTTTATGCTTTG
gga-miR-451	CAGAAACCGTTACCATTACTGAG	GGTCCAGTTTTTTTTTTTTTTTAAACTC
gga-miR-1451-3p	CGTAACTCGCTGCTGTGA	CCAGTTTTTTTTTTTTTTTGCCTCT
gga-miR-29a-3p	CAGTAGCACCATTTGAAATCG	TCCAGTTTTTTTTTTTTTTTAACCGA
gga-miR-130a-3p	GCAGCAGTGCAATATTAAAAGG	GGTCCAGTTTTTTTTTTTTTTTATGC
gga-miR-181a-5p	ACCATCGACCGTTGATTG	TCCAGTTTTTTTTTTTTTTTGGTACA
gga-miR-140-5p	GCAGAGTGGTTTTACCCTATG	GGTCCAGTTTTTTTTTTTTTTTCTAC
gga-miR-17-5p	GCAAAGTGCTTACAGTGCAG	GTCCAGTTTTTTTTTTTTTTTACTACCT
gga-miR-107-3p	GCAGAGCAGCATTGTACAG	GGTCCAGTTTTTTTTTTTTTTTGATAG
gga-miR-33-5p	CGCAGGTGCATTGTAGTTG	GTCCAGTTTTTTTTTTTTTTTGCAA
gga-miR-19b-3p	AGTGTGCAAATCCATGCAA	GGTCCAGTTTTTTTTTTTTTTTCAGT
gga-miR-18a-5p	GCAGTAAGGTGCATCTAGTG	GGTCCAGTTTTTTTTTTTTTTTATCTG

### MiRNA Transfection and Western Blot

To examine the effect of 15 selected SDE miRNAs on NDV replication, DF-1 cells (2 × 10^5^ cells/ml) were seeded on 12-well plates and cultured for 24 h before transfection with indicated miRNA mimics or mimic negative control (mimic-NC) or inhibitors or inhibitor negative control (inh-NC) (Genepharma, Shanghai, China) at the concentration of 100 nM using Lipofectamine 2000 (Invitrogen, CA, USA) following the manufacturer’s instructions. At 18 h after transfection, cells were infected with NDV strain JS5/05 at an MOI of 0.1. After 12 h, cells were lysed in RIPA buffer containing 1 nM phenylmethanesulfonyl fluoride (PMSF). Protein concentrations were determined using the BCA kit. 30ug protein samples of each group were boiled with 6 × SDS loading buffer for 10 min before electrophoresis on 12% sodium dodecylsulfate-polyacrylamide gel electrophoresis (SDS-PAGE) gels, and resolved proteins were transferred onto polyvinylidene difluoride (PVDF) membranes in a transferring buffer (25 mM Tris, 0.2 M glycine and 25% methanol) using electro-transfer. After blocking with 5% skimmed milk, the membranes were incubated with anti-HN and anti-β-actin antibodies, followed by incubation with appropriate HRP-conjugated secondary antibodies (all reagents for Western Blot from Beyotime, Shanghai, China). To examine the effect of gga-miR-451 on YWHAZ expression, DF-1 cells were transfected with gga-miR-451 mimic or mimic-NC or inhibitor or inh-NC. At 18 h after transfection, the cell lysates were subjected to Western Blot analysis using anti-YWHAZ (Sangon Biotech, Shanghai, China) or anti-β-actin antibodies, followed by incubation with appropriate HRP-conjugated secondary antibodies. Blots were developed using an ECL kit. To examine the effect of NDV infection on YWHAZ expression, DF-1 cells were infected or mock infected with different virulence NDV strains (JS5/05, F48E8 Herts/33, LX and La Sota) at an MOI of 0.1. After 12 h, the expression level of YWHAZ was detected by Western Blot.

### Detection of Inflammatory Cytokines and NP Genes by qRT-PCR

DF-1 cells seeded in 12-well plates were transfected with indicated miRNA mimics or mimic-NC or inhibitors or inh-NC or mock treated. At 18 h after transfection, DF-1 cells were infected with JS5/05 at an MOI of 0.1. To detect the effect of gga-miR-451 on NDV-induced inflammatory cytokines, qRT-PCR was performed to measure mRNA expression levels of IFN-β, TNF-α, IL-1β, IL-6, and IL-8 at 12 h after infection. The chicken β-actin gene was selected as a house keeping gene. The total RNA from DF-1 cells was isolated using TRIzol Reagent (TransGen Biotech, Nanjing, China) following the manufacturer’s instructions. The cDNA synthesis and qPCR reaction were performed in one system using TransScript Green One-Step qRT-PCR Super Mix (TransGen Biotech, Nanjing, China) according to the manufacturer’s instructions. To examine the effect of SDE miRNAs on NDV replication, NP gene expression was detected using qRT-PCR after NDV infection. DF-1 cells seeded in 12-well plates were transfected with indicated miRNAs. At 18 h after transfection, DF-1 cells were infected with JS5/05 at an MOI of 0.1. After 12 h, total RNA was extracted and qRT-PCR was performed as described above by using NP primer and chicken β-actin gene served as a house keeping gene. All the primers are listed in Table 2.

**Table 2:** Primers used for inflammatory cytokines, NP gene, and YWHAZ detection.

Gene	Forward primer (5′–3′)	Reverse primer (5′–3′)
IFN-β	TGCACAGCATCCTACTGCTCTTG	GTTGGCATCCTGGTGACGAA
TNF-α	GGACAGCCTATGCCAACAAG	ACACGACAGCCAAGTCAACG
IL-1β	ACCCGCTTCATCTTCTACCG	TCAGCGCCCACTTAGCTTG
IL-6	GGCATTCTCATTTCCTTCT	CTGGCTGCTGGACATTTT
IL-8	CCAAGCACACCTCTCTTCCA	GCAAGGTAGGACGCTGGTAA
NP	GGCAAGGTGCTCTTATATCCCTC	CCTGCGATTACCATGAATCTCTG
*β*-actin	ATTGTCCACCGCAAATGCTTC	AAATAAAGCCATGCCAATCTCGTC
YWHAZ	GGAGCAATCACAACAGGCGT	CAAGGGAACAGGCCTTCTCTGG

### Viral Growth Kinetics Determination

DF-1 cells were transfected with gga-miR-451 mimic or mimic-NC or inhibitor or inh-NC before infection with JS5/05 at an MOI of 0.1.The culture supernatants were collected and replaced with an equal volume of fresh media at different time points (12, 24, 36, 48, and 60 h) after infection. Virus titers of the supernatants were quantified by TCID_50_ on DF-1 cells by the end-point (Reed and Muench, 1938).

### RNA Interference

The YWHAZ-specific small interfering RNAs (siRNAs) were designed and synthesized by Genepharma, Shanghai, China. Sequences of siRNA are as follows: si-YWHAZ sense 5′-GGUGACUAUUACCGUUACUTT-3′, si-YWHAZ antisense 5′-AGUAACGGUAAUAGUCACCTT-3′. The interference efficiency was detected by qRT-PCR using a pair of YWHAZ-specific primers (shown in Table 2). To examine the role of YWHAZ in NDV replication, siRNA duplexes were transfected into DF-1 cells at a final concentration of 50 nM using Lipofectamine 2000, according to the manufacturer’s instructions. After 24 h, JS 5/05 was used to infect DF-1 cells at an MOI of 0.1. The expression levels of IFN-β, TNF-α, IL-1β, IL-6, IL-8, and viral NP gene were measured at 12hpi by qRT-PCR as described above.

### Statistical Analysis

All data are the averages of triplicates and are representative of results from at least three independent experiments. The statistical analysis were performed using one-way analysis of variance (ANOVA) for miRNA, Western Blot, and mRNA expression levels and two-way ANOVA for viral road by GraphPad Prism 5 (GraphPad Software, San Diego, CA, USA). *p* < 0.05 was considered as a significant difference.

## Results

### Newcastle Disease Virus Infection and RNA Extraction From the Samples

In order to investigate the cellular miRNA function during NDV infection, we used NDV strain JS5/05 to infect DF-1 cells. The agarose gel electrophoresis result showed that total RNA of three samples all had high quality with clear 28s, 18s, and 5s straps and no DNA contamination was detected (data not shown). Quality monitoring using Nano Drop and Agilent 2200 Bioanalyzer revealed that all RNA samples met the requirement of library building (Table 3).

**Table 3:** Total RNA quality inspection results.

Group	NanoDrop			AGE^c^	Agilent 2200	
	OD 260/280^a^	OD 260/230^b^	Concentration(ng/μl)		RIN^d^	28S/18S
Mock	1.92	2.17	1273.40	PASS	10.0	3.3
NDV-6 h	1.93	2.19	1088.23	PASS	10.0	3.4
NDV-12 h	1.92	2.20	1065.29	PASS	10.0	3.3

a *OD260/280 reflects the contamination degree of protein and phenol and requires >1.5*.

b *OD 260/230 reflects the contamination degree of salt ions and sugars and the requires >2*.

c *Agarose gel electrophoresis requires clear 28s, 18s, and 5s straps and no DNA contamination*.

d *RNA Integrity Number, an indicator of RNA integrity, requires >7.0*.

### Overview of the Deep Sequencing Results

**Figure 1:**
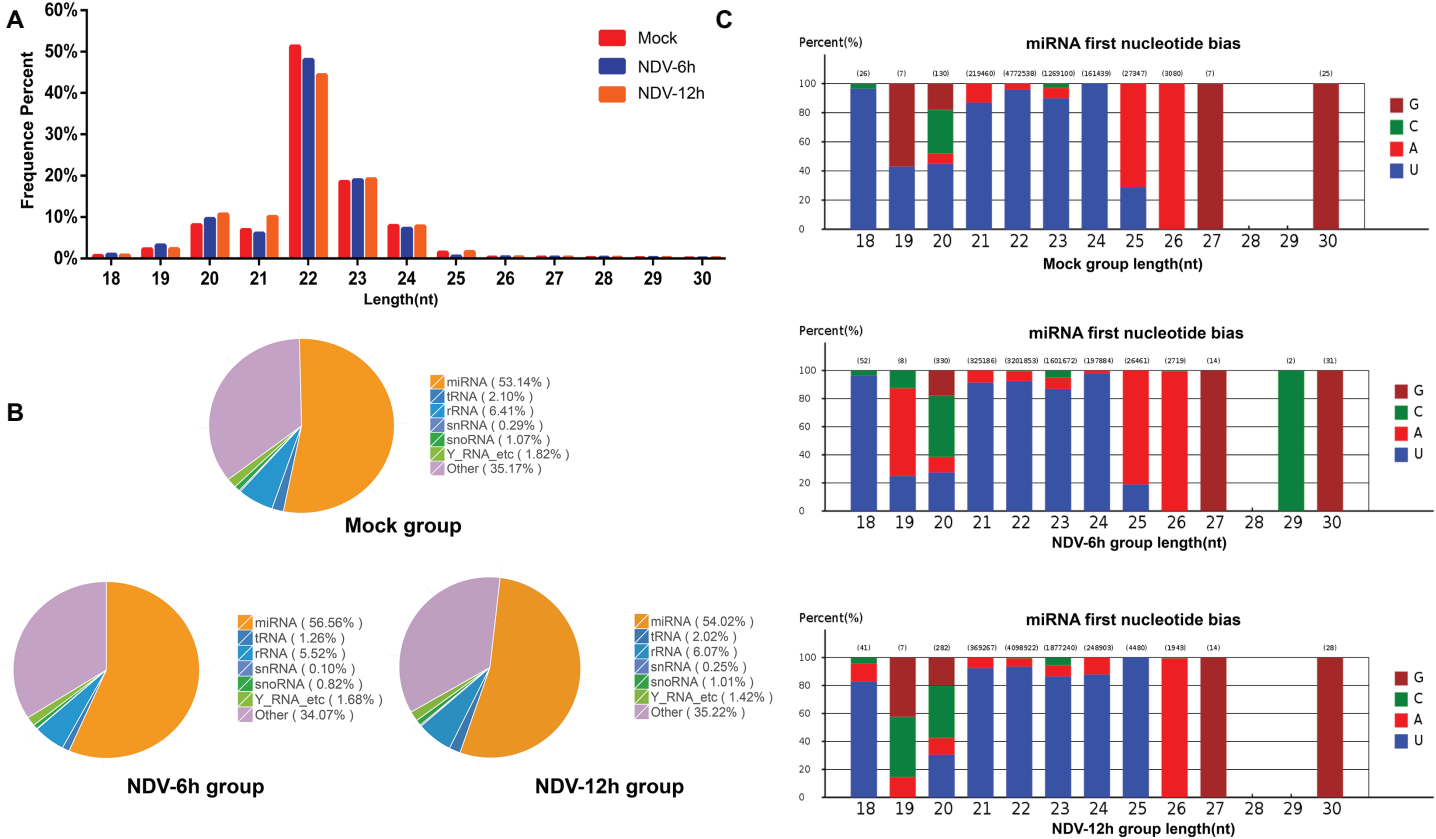
Overview of small RNA deep sequencing data. **(A)** Length distribution of the clean reads measured by deep sequencing. The number of reads is indicated in percentage. Length of most miRNAs in mock, NDV-6 h, and NDV-12 h DF-1 cells was 21–24 nt. **(B)** Pie charts of small RNA-seq showing the percentage of small RNA components in mock, NDV-6 h, and NDV-12 h groups. **(C)** The range in size and base bias at the first position of miRNA identified in mock, NDV-6 h, and NDV-12 h groups. The x-axis indicates miRNAs’ lengths from 18 to 30 nt. The y-axis indicates the percentage of the base bias of miRNAs at the first position of each length.

Three small RNA libraries constructed from mock, NDV-6 h, and NDV-12 h DF-1 cells were sequenced using an Illumina HiSeq 2500 instrument and the summary of small RNAs deep sequencing data is shown in Table 4. About 95% of raw reads were identified as clean reads in each group, which were used for further analysis. All clean reads were classified and annotated into different categories by matching to authoritative databases. Length distributions of the clean reads showed that for all groups, most clean reads were in the range of 21–24 nt (Figure 1A). The first nucleotide bias in identified miRNAs showed a strong preference for “U” at the 5′-end (Figure 1C), which is a characteristic feature of miRNAs. The details of small RNA distribution are shown in Figure 1B. In brief, 53, 56, and 54% of the clean reads corresponded to known miRNAs in mock, NDV-6 h, and NDV-12 h DF-1 cells respectively. The partial of remaining clean reads (<5%) were mapped to other different non-coding RNAs including rRNA, tRNA, snRNA, and snoRNA.

**Table 4:** Summary of small RNAs deep sequencing data.

Categories	Mock		NDV-6 h		NDV-12 h	
	Count	Percentage	Count	Percentage	Count	Percentage
Raw reads	12,960,678	100	12,177,567	100	14,157,277	100
Q30 (%)^a^	12,734,646	98.26	11,941,242	98.06	13,899,687	98.18
Clean reads	12,364,163	95.40	11,668,529	95.82	13,558,883	95.77

a *error rate = 0.10%*.

### Differential Expression Analysis of Known miRNAs in Newcastle Disease Virus-Infected DF-1 Cells

**Figure 2:**
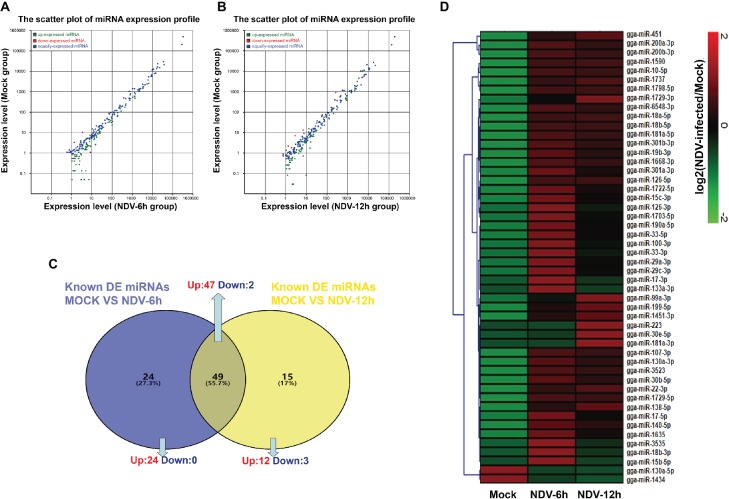
Comparison of expression levels of known miRNAs in different groups. The scatter plot of SDE miRNAs identified in **(A)** (NDV-6 h vs. Mock) and **(B)** (NDV-12 h vs. Mock). The x- and y-axes represent the expression levels of known miRNAs. The red points represent miRNAs with fold changes>2; the gray points represent miRNAs with fold changes between 0.5 and 2; the blue points represent miRNAs with fold changes <0.5. **(C)** Venn diagram showing the number of common differentially expressed miRNAs in NDV-6 h and NDV-12 h groups. Blue and yellow represent the differentially expressed miRNAs of two comparable groups as shown above the circle respectively, and the common parts represent the same SDE miRNAs of these two comparable groups. The numbers in each section indicate the numbers of differentially expressed miRNAs. **(D)** Heatmap of 50 common SDE miRNAs in two comparable groups. Red indicates higher expression, and green indicates lower expression in the three libraries.

To identify known miRNAs expression levels in each group, we aligned the clean reads obtained from each library to mature miRNAs of *Gallus gallus* in miRBase version 21.0 database. A total of 364, 396, and 413 known miRNAs were identified in mock, NDV-6 h, and NDV-12 h DF-1 cells respectively. In order to normalize miRNA quantity, we calculated the RPM values of known miRNAs by computational formula as described above. EdgeR package was used to analyze the significant difference between two groups and calculate *p*. Using │log2 (fold change)│ > 1 and *p*<0.05 as the default threshold for significantly differential expression, 73 miRNAs of NDV-6 h group and 64 miRNAs of NDV-12 h group were significantly differentially expressed (SDE) when compared with those in mock-infected cells respectively. Meanwhile, 50 common SDE miRNAs, including 48 up-regulated and two down-regulated, showed the same expression patterns in NDV-6 h and NDV-12 h groups. Venn diagram of common SDE miRNAs is shown in Figure 2C. The scatter plot of miRNA expression profiles is shown in Figure 2A (NDV-6 h vs. Mock) and Figure 2B (NDV-12 h vs. Mock) and the hierarchical clustering heatmap of common SDE miRNAs is shown in Figure 2D.

### Target Gene Prediction, GO and KEGG Analyses

**Figure 3:**
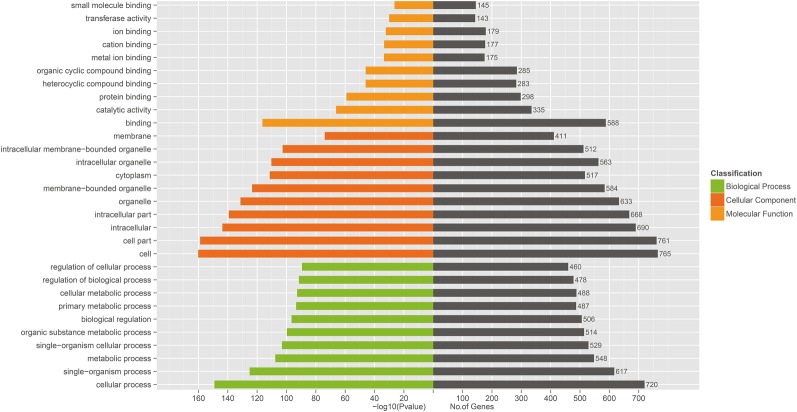
Gene ontology (GO) annotation of predicted target genes from 50 common SDE miRNAs. GO functional analysis utilizing the (GO)::Termfinder software shows that target genes were annotated successfully for 50 common SDE miRNAs, and that these target genes are involved in binding, catalytic activity, cell, cell part, signal-organism process and cell processes. The value of *p* was calculated through hypergeometric distribution and corrected by FDR. GO terms with corrected *p*<0.05 were considered to represent significant enrichment.

**Figure 4:**
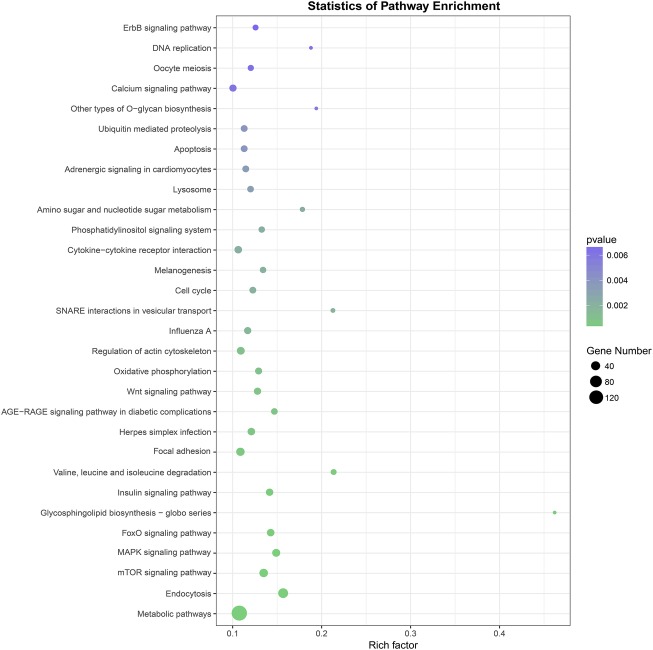
Kyoto Encyclopedia of Genes and Genomes (KEGG) pathway analysis of predicted target genes from 50 common SDE miRNAs. The greener the circle, the more significant the pathway enrichment. The bigger the circle, the higher the number of pathway genes. The results revealed that SDE miRNAs were involved in many important pathways, such as metabolic pathway, endocytosis, mTOR signaling pathway, MAPK signaling pathway, FoxO signaling pathway. *p* was calculated through Fisher’s Exact Test and corrected by FDR. KEGG pathways with corrected *p*<0.05 were considered to represent significant enrichment.

Three prediction algorithms (miRanda, PITA, and RNAhybrid) were used to predict the potential target genes of 50 common SDE miRNAs. Finally, 4,011 target genes were chosen for the next functional analyses. GO annotation of the target genes of SDE miRNAs suggested that SDE miRNAs were involved in cellular process, signal-organism process, metabolic process, and other processes (Figure 3). To analyze the regulatory roles of common SDE miRNAs, KEGG pathway enrichment was carried out and the results revealed that these common SDE miRNAs were involved in many important pathways, such as metabolic pathway, endocytosis, mammalian target of rapamycin (mTOR) signaling pathway, mitogen-activated protein kinase (MAPK) signaling pathway, and forkhead box O (FoxO) signaling pathway (Figure 4).

### Validation of miRNA Expression by Stem-Loop qRT-PCR

**Figure 5:**
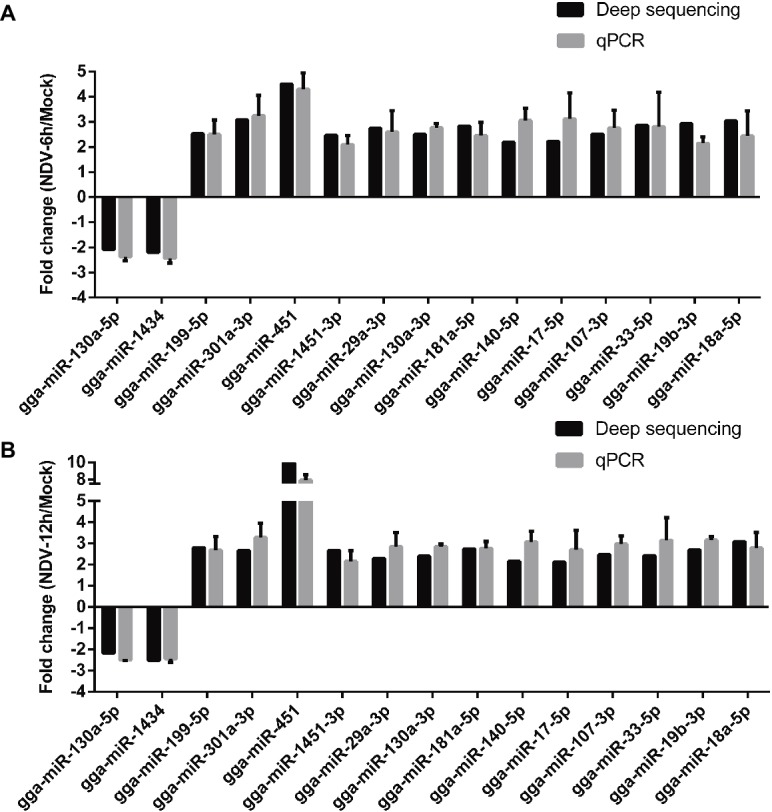
qRT-PCR validation of deep sequencing result. DF-1 cells were infected with JS5/05 at an MOI of 0.1. MiRNAs of mock, NDV-6 h, and NDV-12 h DF-1cells were extracted at 0, 6, and 12 hpi, respectively. Expression levels of 15 selected common SDE miRNAs were measured by qRT-PCR. **(A)** The result of NDV-6 h/Mock and **(B)** the result of NDV-12 h/Mock. MiRNAs’ expression levels were normalized to 5s rRNA levels using the 2^−ΔΔCt^ model. Average data from three independent experiments (mean ± SD) were presented.

To validate deep sequencing data, the expression levels of 15 randomly selected miRNAs from 50 common SDE miRNAs were measured by stem-loop qRT-PCR. The results showed that expression levels of all the 15 selected miRNAs determined by qRT-PCR were consistent with those measured by deep sequencing, indicating the reliability of the deep sequencing data. As shown in Figure 5, two miRNAs (gga-miR-130a-5p, gga-miR-1434) were significantly down-regulated, and 12 miRNAs (gga-miR-199-5p, gga-miR-301a-3p, gga-miR-451, gga-miR-130a-3p, gga-miR-181a-5p, gga-miR-140-5p, gga-miR-17-5p, gga-miR-107-3p, gga-miR-33-5p, gga-miR-19b-5p, and gga-miR-18a-5p) were significantly up-regulated in both NDV-6 h (Figure 5A) and NDV-12 h (Figure 5B) groups when compared with mock group.

### Effect of miRNAs on Newcastle Disease Virus Replication

**Figure 6:**
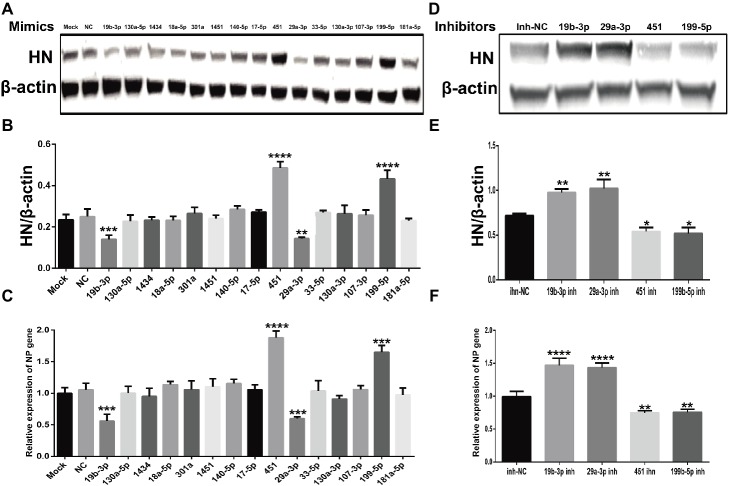
The effect of common SDE miRNAs on the expression of viral HN protein and NP gene. DF-1 cells were transfected with indicated miRNA mimics or mimic-NC or inhibitors or inh-NC at the concentration of 100 nM. At 18 h after transfection, cells were infected with JS5/05 at an MOI of 0.1 for 12 h. The cell lysates were prepared for Western Blot using anti-HN antibody, and the β-actin expression was used as an internal control. Meanwhile, total RNA was extracted for viral NP gene detection by qRT-PCR using β-actin as an internal control. The band density of HN protein in mimics **(A)** and inhibitors **(D)** groups were quantitated by densitometry. The relative levels of HN expression were calculated as follows: band density of HN/band density of β-actin in the same sample and are shown in **(B,E)** respectively. The relative expression levels of NP gene in mimics and inhibitors groups are shown in **(C,F)** respectively. Data are from three independent experiments and presented as mean ± SD. *stands for *p* < 0.05, **stands for *p* < 0.01, ***for *p* < 0.001, and ****for *p* < 0.0001.

As important regulators post transcription, miRNAs have been reported to be involved in the conflict between host cells and viral infection and replication (Trobaugh and Klimstra, 2017). To investigate the roles of these common SDE miRNAs in NDV replication, we transfected indicated miRNA mimics or mimic-NC or inhibitors or inh-NC into DF-1 cells followed by NDV infection and viral HN protein and NP gene were detected by Western Blot and qRT-PCR, respectively. As shown in Figures 6A–C, overexpression of gga-miR-451 and gga-miR-199-5p enhanced the expression of viral HN protein and NP gene, while their inhibition significantly reduced the expression of viral HN protein and NP gene. On the contrary, overexpression of gga-miR-19b-3p and gga-miR-29a-3p suppressed the expression of viral HN protein and NP gene, while their inhibition showed an opposite effect on viral HN protein and NP gene (Figures 6D–F). These results clearly establish the role of gga-miR-451 and gga-miR-199-5p as two pro-viral factors in DF-1 cells’ response to NDV infection, while gga-miR-19b-3p and gga-miR-29a-3p as two anti-viral factors.

### Gga-miR-451 Inhibits Newcastle Disease Virus-Induced Inflammatory Cytokine Expression to Enhance Virus Replication *via* Targeting YWHAZ

**Figure 7:**
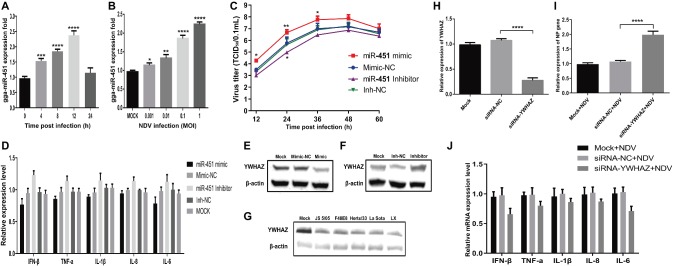
Gga-miR-451 negatively regulates NDV-induced inflammatory cytokines to enhance NDV replication *via* targeting YWHAZ. Gga-miR-451 expression is up-regulated after NDV infection **(A,B)**. DF-1 cells were infected with NDV at an MOI of 0.1 for indicated times **(A)** or at indicated MOIs for 12 h **(B)**, and then the expression levels of gga-miR-451 were measured by qRT-PCR. **(C)** Analysis of the effect of gga-miR-451 on NDV replication by TCID_50_ assay. **(D)** DF-1 cells were transfected with gga-miR-451 mimic or mimic-NC or inhibitor or inh-NC or mock transfected, then infected with JS5/05 at an MOI of 0.1. The relative mRNA expression levels of IFN-β, TNF-α, IL-1β, IL-6, and IL-8 were analyzed by qRT-PCR at 12 hpi using β-actin as an internal control. **(E,F)** The expression relationship between gga-miR-451 and YWHAZ was verified by Western Blot assay in DF-1 cells. **(G)** The expression levels of YWHAZ protein after being infected or mock infected with different virulence NDV strains (JS5/05, F48E8, Herts/33, LX and La Sota) were detected using Western Blot. **(H)** The interference efficiency of siRNA-YWHAZ was detected at 24 h after transfection by qRT-PCR. **(I)** The viral NP gene expression was measured after transfection of siRNA-YWHAZ by qRT-PCR. **(J)** NDV-induced inflammatory cytokines after knockdown of YWHAZ were detected using qRT-PCR. Data are from three independent experiments and presented as mean ± SD. *stands for *p* < 0.05, **for *p* < 0.01, ***for *p* < 0.001, and ****for *p* < 0.0001.

To further explore the effect of gga-miR-451 on NDV replication, we firstly measured the dynamic expression pattern of gga-mir-451 in DF-1 cells after NDV infection. As shown in Figure 7A, the expression level of gga-miR-451 was significantly increased and reached the peak at 12 h after NDV infection, then decreased and returned to normal. In addition, dose-dependent increase between gga-miR-451 and NDV infection is revealed in Figure 7B. Viral growth kinetics shown in Figure 7C indicates that gga-miR-451 mimic could promote NDV replication while gga-miR-451 inhibitor showed a weak ability for inhibiting NDV replication. To detect the effect of gga-miR-451 on NDV-induced inflammatory cytokine, we detected the mRNA levels of IFN-β, TNF-α, IL-1β, IL-6, and IL-8 after NDV infection by qRT-PCR. As shown in Figure 7D, overexpression of gga-miR-451 reduced the expression levels of all the five detected cytokines, while inhibition of gga-miR-451 showed an opposite effect. These data strongly indicate that gga-miR-451 reduces the production of inflammatory cytokines in NDV-infected DF-1 cells. In order to confirm the interaction between gga-miR-451 and YWHAZ in DF-1 cells, we detected YWHAZ expression after miRNA transfection or different virulence NDV strains infection using Western Blot. As shown in Figures 7E,F, overexpression of gga-miR-451 resulted in a significant reduction in YWHAZ while inhibition of gga-miR-451 increased the expression of YWHAZ, which is consistent with previous studies and indicated that YWHAZ is a direct target of gga-miR-451in DF-1 cells. Moreover, the expression levels of YWHAZ in all NDV-infected DF-1 cells were significantly decreased in a parallel degree as shown in Figure 7G. These results suggested that the up-regulated gga-miR-451 due to NDV infection inhibits YWHAZ production and this interaction between gga-miR-451 and YWHAZ is universal among different virulence NDV strains infection *in vitro*.

Furthermore, to examine the role of YWHAZ in NDV replication, YWHAZ-specific siRNAs were used for the suppression of YWHAZ with an interference efficiency of about 70% at 24 h after transfection (Figure 7H). The expression levels of inflammatory cytokines and viral NP gene in siRNA-NC and siRNA-transfected DF-1 cells were measured by qRT-PCR after NDV infection. As a result, knockdown of YWHAZ by RNA interference markedly inhibits NDV-induced expression of inflammatory cytokines in DF-1 cells (Figure 7J). In contrast, NDV replication was significantly enhanced by knockdown of YWHAZ (Figure 7I), similar to the effect of gga-miR-451 on NDV replication. Therefore, these results indicated that that gga-miR-451 can inhibit NDV-induced inflammatory cytokines, thereby promoting NDV replication in DF-1 cells *via* targeting YWHAZ.

## Discussion

Since the regulatory function of first miRNA, lin-4, was demonstrated in 1999 (Feinbaum and Ambros, 1999; Olsen and Ambros, 1999), the ubiquitously regulating gene expression function of miRNAs has already become a hot research area in the past two decades. Recent studies have revealed that host miRNAs can affect virus infection and replication and their expression levels are often deregulated by viral infections. For some paramyxoviruses, such as RSV, HeV, and Nipah virus, critical role of cellular miRNAs in host defense against virus infection has already been characterized. However, little is known about the cellular miRNAs expression profile and the molecular mechanism of the regulatory function of miRNA after NDV infection in avian cells. In this study, we evaluated the miRNA expression profile in DF-1 cells in response to NDV infection. DF-1 is a continuous cell line of chicken embryo fibroblasts that is commonly used for studies of avian viruses. According to deep sequencing results, 364, 396, and 413 known miRNAs were identified in mock, NDV-6 h, and NDV-12 h DF-1 cells respectively. Fifty SDE miRNAs (two down-regulated and 48 up-regulated) showed the same expression patterns in NDV-6 h and NDV-12 h groups when compared with those in mock group. Compared with a previous study (Jia et al., 2018), some miRNAs were also differentially expressed in NDV-infected chicken embryonic tissues. For instance, the expression of gga-miR-126-5p, gga-miR-30b-5p, gga-miR-30e-5p, gga-miR-200b-3p, and gga-miR-126-3p is up-regulated according to our deep sequencing results, which is consistent with these in different NDV-infected chicken embryonic tissues. However, gga-miR-199-5p and gga-miR-140-5p show opposite expression patterns between our results and previous report. These inconsistent expression patterns of miRNAs between NDV-infected DF-1 cells and chicken embryonic tissues may be due to the tissue specificity and temporal expression of miRNAs.

Although, most of the functions of these SDE miRNAs and the mechanisms by which they act are still unknown, some have already been reported to play roles in the interaction between host and virus. The expression of gga-miR-130a-3p and gga-miR-181a-5p were reduced in Marek’s disease virus (MDV)-infected tissues. Further functional studies revealed that gga-miR-130a and gga-miR-181a showed an inhibitory effect on MDV-transformed lymphoid cells (MSB1) proliferation and migration *via* targeting homeobox A3 (HoxA3) and v-myb myeloblastosis viral oncogene homolog-like 1 (MYBL1), respectively (Peng et al., 2015; Han et al., 2016). Moreover, gga-miR-181a-5p is important for the immune response to AIV (Peng et al., 2015). To date, miR-223 has been demonstrated to be involved in many types of inflammatory diseases, cancers, autoimmune diseases, and other pathological processes. Moreover, up-regulated miR-223 in VSV-infected macrophages regulated the type I IFN production in the antiviral innate immunity *via* directly targeting FoxO3. Deep sequencing analysis of MG-infected lungs of specific pathogen-free chicken embryos revealed that gga-miR-99a is down-regulated (Zhao et al., 2017a). Further study showed that gga-miR-99a targeted the SWI/SNF-related, matrix-associated, actin-dependent regulator of chromatin, subfamily a, member 5 (SMARCA5) to regulate *Mycoplasma gallisepticum* infection by depressing cell proliferation in chickens (Zhao et al., 2017b). In the current study, we found that gga-miR-130a-3p, gga-miR-181a-5p, gga-miR-223, and gga-miR-99a-5p were all up-regulated upon NDV infection. These results strongly indicated a possibility that these miRNAs likely participate in host-virus interaction in DF-1 cells.

Mounting evidences suggest that miRNAs can coordinate host defense against viral infection. On one hand, host miRNAs can inhibit viral replication and regulate host antiviral immunity (Zhu et al., 2014). On the other hand, miRNAs can also be manipulated by viruses to facilitate viral replication (Wu et al., 2013; Chang et al., 2015). To explore the functional miRNAs, we examined the effect of common SDE miRNAS on NDV replication. Our study revealed that gga-miR-451 and gga-miR-199b-5p could promote NDV replication, while gga-miR-19b-3p and gga-miR-29a-3p could inhibit NDV replication. To our knowledge, this contrasting effects of cellular miRNAs on viral replication contribute to maintain homeostasis. However, whether there is a synergy or antagonism between gga-miR-451 and 19b-3p remains to be further studied.

Further functional study of gga-miR-451 revealed that overexpression of gga-miR-451 could significantly promoted NDV replication compared with the control group. However, inhibitors group of gga-miR-451 showed no significant decrease except for 12 hpi. This unexpected result may be due to the very low level of endogenic gga-miR-451 expression in DF-1 cells. Moreover, cells have very strong compensatory mechanisms (Wang et al., 2019). Each gene may be targeted by hundreds of miRNAs, and each miRNA may regulate hundreds of genes (Trobaugh and Klimstra, 2017). Therefore, this unexpected result might also be due to the presence of the large number of cellular regulatory factors which can offset or mask this inhibitory effect on viral proliferation. Multiple researches of miR-451 have revealed that miR-451 can directly bind YWHAZ in mammals and avians (Yu et al., 2010; Joshi et al., 2012; Rosenberger et al., 2012; Li et al., 2015; Wang et al., 2017; Zhao et al., 2018). YWHAZ is known as a negative regulator of Zinc Finger Protein 36 (ZFP36), an RNA binding protein that targets AU-rich mRNAs such as TNF-α, CCL3, and IL-6 for degradation (Rosenberger et al., 2012). In our study, the expression of YWHAZ was inversely correlated with the expression of gga-miR-451, indicated that gga-miR-451 also directly targets YWHAZ in DF-1 cells. Different virulence NDV strains could suppress the expression of YWHAZ in a similar level, indicated a virulence-independent effect of YWHAZ on NDV replication. We also found that overexpression of gga-miR-451 suppressed NDV-induced inflammatory cytokines, which was similar with knockdown of YWHAZ by siRNA. Therefore, gga-miR-451 may suppress YWHAZ to decrease the expression of inflammatory cytokines and consequently enhance NDV replication *in vitro*. However, the specific functions of most differentially expressed miRNAs still need to be further explored.

## Author Contributions

YC was responsible for experiment design, data analysis, and writing the manuscript. WL, HX, YD, HC, SZ, and YP were responsible for performing experiments. JL, JH, XW, MG, and XWL were responsible for suggestion during the experiments performance. ZH, SH, and XFL were responsible for revising the manuscript. All authors read and approved the final manuscript.

### Conflict of Interest Statement

The authors declare that the research was conducted in the absence of any commercial or financial relationships that could be construed as a potential conflict of interest.

## Footnotes

^1^http://www.mirbase.org/

^2^http://rfam.xfam.org

^3^http://pirnabank.ibab.ac.in/

^4^https://www.microrna.org/

^5^https://genie.weizmann.ac.il/pubs/mir07/

^6^https://bibiserv.cebitec.uni-bielefeld.de/rnahybrid

^7^http://www.genome.jp/kegg/
